# Comparative Evaluation of Canal Centering Ability and Transportation of MiniKUT and TruNatomy Rotary File Systems in Curved Root Canals: A Cone-Beam Computed Tomographic Study

**DOI:** 10.1055/s-0045-1812310

**Published:** 2025-11-07

**Authors:** Hemant R. Chourasia, Mohammed Y. Tarrosh, Amani Alfaifi, Kawther Alhazmi, Fatima Khormi, Ahmad H. Jabali

**Affiliations:** 1 Division of Endodontics, Department of Restorative Dental Sciences, College of Dentistry, Jazan University, Jazan, Saudi Arabia; 2 College of Dentistry, Jazan University, Saudi Arabia

**Keywords:** centering ability, curved canal, cone-beam computed tomography, MiniKUT, root canal transportation, TruNatomy

## Abstract

**Objectives:**

The current in vitro analysis was designed to compare the centering ability and transportation of MiniKUT and TruNatomy files on the curved mesiobuccal root of the mandibular first molars using cone-beam computed tomography (CBCT).

**Materials and Methods:**

Thirty extracted mandibular first molars were randomly divided into two groups. In the first group, root canal instrumentation was performed by MiniKUT, and in the second group, TruNatomy rotary files were used. CBCT analysis of pre- and post-instrumented teeth was performed to check the canal transportation and centering ability at distances of 3, 6, and 9 mm from the root apex with the help of a prevalidated formula.

**Statistical Analysis:**

Descriptive statistics were performed to calculate the mean and standard deviation. One-way ANOVA followed by post hoc Bonferroni correction was used to check the association at different levels. Pearson's correlation analysis was performed to assess the relationship between the groups.

**Results:**

A significant association was reported with centering ability at 3, 6, and 9 mm ( *p* = 0.001) across the groups. However, no significant association was reported with canal transportation at 3, 6, and 9 mm ( *p* = 0.16) among both groups. TruNatomy showed higher transportation as compared to MiniKUT at 3 mm from the apex. Overall, the result suggests both files are safer to use in curved mandibular canals.

**Conclusion:**

Both MiniKUT and TruNatomy files demonstrated minimal root canal transportation and maintained canal shape at 3, 6, and 9 mm from the apex. These characteristics suggest that both systems are safe and effective for the instrumentation of curved mandibular canals.

## Introduction

The success of endodontic treatment relies mainly on effective root canal preparation, which involves cleaning, shaping, and disinfecting the root canal system while maintaining its original anatomy. One of the most critical challenges in endodontics is the management of curved root canals, where procedural errors such as transportation, ledging, and perforations can compromise treatment outcomes. 1 2 In a study by Sunildath et al, 3 approximately 49% of curved root canals exhibited varying degrees of apical transportation following instrumentation. The restoring forces generated by endodontic file systems tend to straighten the natural curvature of the root canal, thereby contributing to canal transportation. 3

Nickel-titanium (NiTi) rotary instruments have transformed root canal preparation by providing superior flexibility and cutting efficiency compared to stainless steel files. 4 However, challenges such as canal transportation and other iatrogenic errors remain, particularly in curved canals, which can compromise treatment outcomes. 5 To overcome these limitations, newer NiTi systems with advanced metallurgy and design modifications, including heat-treated alloys, off-centered cross-sections, and reduced tapers, have been introduced. 6 Controlled memory NiTi instruments, in particular, offer improved flexibility, fatigue resistance, and torsional strength, thereby reducing canal aberrations and maintaining the original canal anatomy more effectively than conventional NiTi files. 7 8

Within this context, two novel systems, TruNatomy (Dentsply Sirona, United States) and MiniKUT (PlanB Dental, Germany), have been specifically developed with the philosophy of conservative canal preparation. TruNatomy employs a slim core, off-centered parallelogram, and regressive design to preserve dentin and reduce shaping stress, while MiniKUT features a unique flute geometry that enhances debris removal and navigates complex curvatures with minimal torsional stress. 9 10 11 12 13 14 15 TruNatomy files are manufactured using a proprietary heat-treated NiTi alloy that provides enhanced flexibility and resistance to cyclic fatigue, while MiniKUT files are produced using advanced machine and heat treatment NiTi technology, designed to maintain canal anatomy and reduce the risk of transportation in curved canals. 15 Previous studies have shown TruNatomy to produce less canal transportation than ProTaper Gold and HyFlex EDM systems. 10 16 17 However, there is limited evidence directly comparing MiniKUT and TruNatomy, despite both being marketed as minimally invasive systems designed to optimize shaping while preserving canal anatomy. This gap highlights the need for a comparative evaluation of their canal centering ability and transportation in curved root canals, which is the focus of the present study. 18

Cone-beam computed tomography (CBCT) has emerged as a valuable tool for evaluating root canal preparation accuracy. It allows three-dimensional assessment of canal transportation, centering ability, and dentin removal. 14 19 Unlike traditional radiographic methods, CBCT provides high-resolution cross-sectional images that enable precise measurements of pre- and post-instrumentation canal anatomy. 14 Although micro-CT offers superior spatial resolution and continuous 3D data acquisition, its application is limited by high cost, restricted availability, and time-consuming scanning and reconstruction processes. 20 Several studies have utilized CBCT to compare different NiTi systems, but none have focused on the newer TruNatomy and MiniKUT files. 7 21 22

To date, no studies have investigated and compared the centering ability and transportation of TruNatomy and MiniKUT rotary files. Therefore, the current in vitro analysis was designed to compare the centering ability and transportation of TruNatomy and MiniKUT files on curved mesiobuccal (MB) root canals of mandibular first molar by employing CBCT. Specific parameters include canal transportation and centering ability at different root levels (3, 6, and 9 mm from the apex). By assessing these outcomes, this study seeks to provide clinical insights into the efficacy of these two systems, helping clinicians make evidence-based decisions for endodontic therapy in curved or anatomically challenging canals. The null hypothesis generated for the current analysis was that there is no difference in centering ability and root canal transportation between the two novel rotary systems.

## Materials and Methods

The ethical approval for this in vitro study was obtained from the institutional ethics committee, and the study was conducted between March 2025 and June 2025.

### Tooth Selection and Sample Size Calculations

Following ethical approval, 55 intact human mandibular first molars, extracted for compromised periodontal health, were collected. Informed consent was obtained from patients to utilize their extracted teeth for research and educational purposes. The teeth were then stored and handled according to the guidelines presented by the Centers for Disease Control and Prevention. 23 The included samples comprised teeth with mature apices, a root length of 19 to 22 mm, no previous endodontic treatment, apical diameters compatible with a #10 K-file at working length, and 20 to 30 degrees of mesial root curvature, based on Schneider's criteria. The canal curvature was carefully balanced across the two groups to ensure comparable anatomical conditions for the MiniKUT and TruNatomy files. However, teeth with fractured root canals, microcracks, calcified canals, and mesial root curvature greater than 30 degrees were excluded.

The sample size was calculated using the G power sample size formula ( https://www.calculator.net/sample-size-calculator.html ). A minimum of 10 teeth per group was required to obtain 80% power and a type 1 error of 0.05 for approximate results.

Teeth were radiographed in a mesiodistal direction, and the curvature of the MB canal was measured based on Schneider's method. 22 In this method, one line is drawn parallel to the long axis of the canal in the coronal third and another from the apical foramen to the point of initial curvature; the angle formed at their intersection represents the degree of curvature ( Fig. 1 ). 22 Following exclusion criteria and radiographical analysis, a total of 10 teeth were excluded. Finally, in the current in vitro analysis, 30 mandibular molars with 20 to 30 degrees of mesial root curvature were selected.

**Fig. 1:**
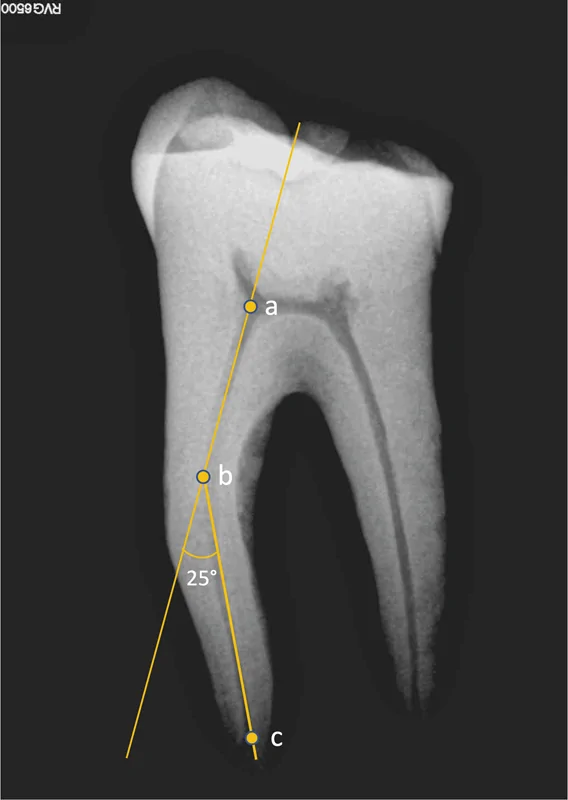
Radiograph of a sample tooth with Schneider's method applied, demonstrating a root canal curvature of 25 degrees.

### Sample Preparation

The sample teeth were randomly assigned to two experimental groups ( *n* = 15 each group): Group 1—MiniKUT (PlanB Dental) and Group 2—TruNatomy (Dentsply Sirona). A pre-instrumentation CBCT scan (RAYPreMiere, Ray America Inc., United States) at 90 kV and 7 mA, 0.2 mm ^3^ voxel size, 15.8 seconds exposure time, and 140 × 100 field of view was obtained to assess the initial root canal morphology. Following this, the images were analyzed using the OnDemand3D Communicator software (version 1.0.11). A single operator, with more than 20 years of experience in endodontics, performed the endodontic procedures on all the included samples. Two endodontists interpreted the images, and the intra-rater reliability was assessed using Cohen's kappa statistics, with results ranging between 0.8 and 0.9.

### Access Cavity Preparation

Access cavities were prepared in all sample teeth, using a round diamond and tapered fissure burs with a high-speed handpiece under air–water spray, and a #10 K-file (Dentsply Maillefer, Switzerland) was used to locate the MB canal. The working length was established at 0.5 mm short of the apex. To maintain uniformity, the teeth were decoronated at 16 mm from the apex using a diamond disc. The teeth specimens were embedded in dental wax sheets and molded using prefabricated rubber molds. The teeth were then divided into two groups.

For Group 1 (MiniKUT), a glide path was established using manual K-files (size #10) to confirm canal patency up to the working length. Subsequently, files from the MiniKUT procedure pack for small canals (15/07, 15/03, and 25/03) were used at 800 rpm and a torque of 2.00 Ncm according to the manufacturer's recommendations. The canal was frequently irrigated with 5.25% sodium hypochlorite (NaOCl) using a 27-gauge needle attached to a syringe after each file insertion.

In Group 2 (TruNatomy), manual K-files (size #10) were used to confirm canal patency and the working length. The TruNatomy assorted files (O modifier 20/08, glider 17/02, small 20/04, prime 26/04, and medium 36/03) were used at 500 rpm with 1.5 Ncm torque as per the manufacturer's recommendations. After each file insertion, the canal was thoroughly irrigated with 5.25% NaOCl using a syringe equipped with a 27-gauge needle.

A 1-mL solution of 17% ethylenediaminetetraacetic acid (EDTA) was used for 1 minute to effectively chelate and remove the inorganic component of the smear layer, followed by a final irrigation with 5.25% NaOCl to dissolve organic tissue remnants. 24 25 To evaluate the changes in the canal morphology, post-instrumentation CBCT scans were obtained using the same exposure parameters as the pre-instrumentation scans (90 kV and 7 mA, 0.2 mm ^3^ voxel size, 15.8 s exposure time, and 140 × 100 field of view).

The distance from the internal canal wall of the MB canal to the external root surface was measured at three standardized levels: 3, 6, and 9 mm from the apex. These levels correspond to the apical, middle, and coronal thirds of the root canal and provide a standardized, reproducible assessment of shaping at different anatomical regions. 26 27 Moreover, canals are highly susceptible to iatrogenic mishaps such as transportation at 3, 6, and 9 mm from the apex. 25

### Canal Transportation and Centering Ability Calculation

Pre- and post-instrumentation CBCT scans ( Fig. 2 ) were analyzed by measuring the shortest distances from the root periphery to the unprepared and prepared canal walls in mesial and distal directions ( Fig. 3 ). Canal transportation was calculated using the formula of Gambill et al 28 : *(a1 − a2) − (b1 − b2)* , where

**Fig. 2:**
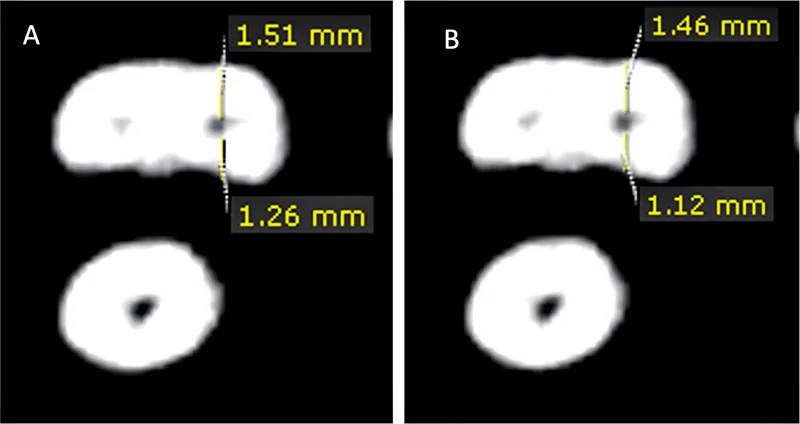
Axial CBCT sections showing pre-instrumentation ( **A** ) and post-instrumentation ( **B** ) measurements of root canal transportation (the OnDemand3D Communicator software, version 1.0.11).

**Fig. 3:**
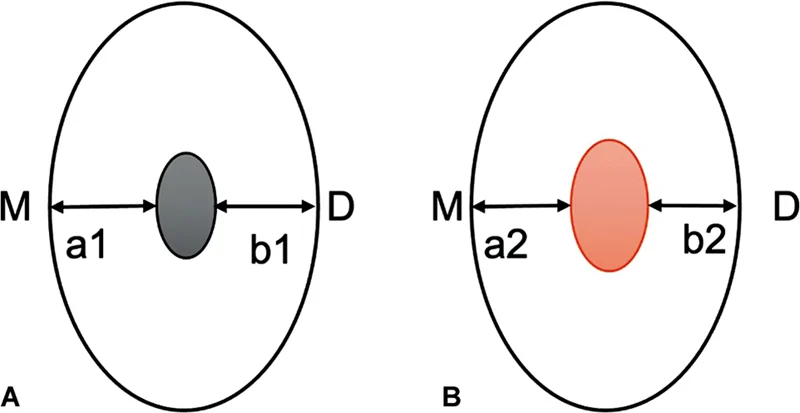
Schematic representation of measurements of the canal for transformation and centering ability. ( **A** ) Pre-instrumentation; ( **B** ) post-instrumentation (the figure was created on Adobe Photoshop version 25.0).

- **a1** = mesial root edge to mesial uninstrumented canal wall.
- **b1** = distal root edge to distal uninstrumented canal wall.
- **a2** = mesial root edge to mesial instrumented canal wall.
- **b2** = distal root edge to distal instrumented canal wall.

For centering ability, pre- and postoperative cross-sectional views of the root were analyzed along with the mean centering ratio. The following formula was used to determine the centering ability: (a1 − a2) / (b1 − b2) or (b1 − b2) / (a1 − a2). According to this formula, the value of a indicates 1, which means perfect canal centering, and the values near 0 indicate that the ability of the instrument to maintain canal centering decreases.

## Statistical Analysis

The data were extracted and imported into Microsoft Excel version 2501 (IBM, Microsoft Inc., United States). Statistical analysis was performed using SPSS (Statistical Package for the Social Sciences; version 20.0, Chicago, Illinois, United States). Descriptive statistics for canal transportation and centering ability were presented as means and standard deviations. The one-way ANOVA was used to compare the canal transportation and centering ability of two different rotary instruments at various levels. The post hoc Bonferroni test was used to determine which of the two groups is responsible for the statistical significance. The Pearson correlation test was used to determine the linear correlation between transportation and centering ability for both groups. The statistical significance was established at 0.05.

## Results

Table 1 summarizes the mean canal transportation (±standard deviations) and coefficients of variation at 3, 6, and 9 mm from the apex for MiniKUT and TruNatomy rotary file systems. At the 3-mm level, both systems showed transportation close to zero. MiniKUT had a slightly negative mean value (−0.0033 ± 0.1492), while TruNatomy showed a slightly positive deviation (0.0333 ± 0.1196). At the 9-mm level, MiniKUT displayed a higher negative deviation (−0.0773 ± 0.1612) compared to TruNatomy (−0.0526 ± 0.1472). The one-way ANOVA revealed no statistically significant difference between the two systems in terms of canal transportation at different root levels ( *p* = 0.165), suggesting that both systems performed similarly in maintaining root canal transportation.

**Table 1:** A comparison between two different groups for evaluating canal transportation at three different levels (3, 6, and 9 mm) using a one-way ANOVA test

Root levels							
Groups	3 mm		6 mm		9 mm		*p* -Value (ANOVA)
	SD	Coefficient of variance	SD	Coefficient of variance	SD	Coefficient of variance	0.165 a
MiniKUT	0.1492	0.743	0.2019	0.433	0.1612	0.438	
TruNatomy	0.1196	0.464	0.2510	0.668	0.1472	0.665	

Abbreviations: SD, standard deviation; ANOVA, analysis of variance.

a *p* > 0.05.

Table 2 presents the mean centering ability of canal values (in millimeters) along with standard deviations and coefficients of variation at three levels of the root canal: 3, 6, and 9 mm from the apex for both MiniKUT and TruNatomy rotary file systems. At the 3-mm level, MiniKUT showed higher centering ability (mean = 0.1855 ± 0.1485 mm) compared to TruNatomy (mean = 0.1081 ± 0.0552 mm), with a notably higher coefficient of variation (1.893 vs. 0.069), suggesting more variability in the performance of MiniKUT at this level. At the 9-mm level, MiniKUT again showed a higher mean centering ability (0.5298 vs. 0.4992 mm), although TruNatomy presented a higher coefficient of variation (0.770), indicating greater inconsistency in canal shaping at this level. The ANOVA test revealed a statistically significant difference between the two systems across the three levels ( *p* = 0.001), indicating that the type of file system significantly influenced the extent of centering ability.

**Table 2:** A comparison between two different groups for evaluating centric ability at three different levels (3, 6, and 9 mm) using a one-way ANOVA test

Root levels							
Groups	3 mm		6 mm		9 mm		*p* -Value (ANOVA)
	Mean (SD)	Coefficient of variance	Mean (SD)	Coefficient of variance	Mean (SD)	Coefficient of variance	0.001 a
MiniKUT	0.1855 (0.1485)	1.893	0.1112 (0.0715)	0.411	0.5298 (0.3421)	0.295	
TruNatomy	0.1081 (0.0552)	0.069	0.1212 (0.0602)	0.684	0.4992 (0.2103)	0.770	

Abbreviations: SD, standard deviation; ANOVA, analysis of variance.

a *p* > 0.05.

Fig. 4 represents a violin plot for post hoc analysis of centering ability among both groups. The shape of each violin represents the distribution of data points. Wider sections indicate a higher density of values, while narrower sections show fewer data points. The individual dot points show raw data values. At 3 mm, Group 1 has a wider distribution, suggesting more variability in centering ability, while Group 2 shows a more compact shape, meaning it maintained a more consistent centering ability. This could indicate that Group 2's file system is better at maintaining canal shape in the apical third. At 6 mm, both groups show similar distributions, meaning they performed comparably at this level. Finally, at 9 mm, Group 1 has a much wider spread, with some outliers showing very high centering ability, and Group 2 remains more compact, again indicating more consistency in shaping.

**Fig. 4:**
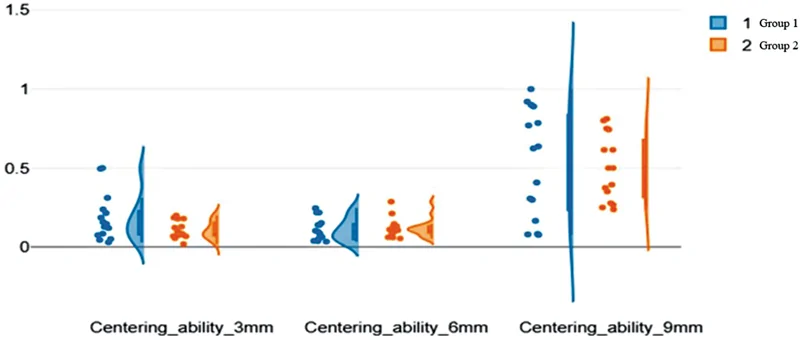
The violin plot represents the difference in centering ability between two groups, where blue color represents group 1 (MiniKUT) and orange color represents group 2 (TruNatomy).

Pearson's correlation ( Table 3 ) revealed a significant positive association at 3 mm in Group 1 ( *r* = 0.579, *p* = 0.024), while no significant correlations were found at other levels ( *p* > 0.05) for either group. Group 2 showed no significant correlations at any level (all *p* > 0.05). These results suggest instrumentation technique in Group 1-influenced centering ability primarily in the coronal third (3 mm), whereas Group 2 maintained consistent performance throughout all canal levels without significant correlations. Overall, both files are proven to be good for biomechanical preparation for curved molar roots.

**Table 3:** Correlation between two different groups using a Pearson correlation analysis

	Group 1		Group 2	
	*r* -Value	*p* -Value	*r* -Value	*p* -Value
**At 3 mm**	0.579	0.024 a	−0.292	0.290
**At 6 mm**	0.137	0.626	0.369	0.176
**At 9 mm**	−0.051	0.858	−0.288	0.298

a Pearson's correlation coefficient: *p* < 0.05, significant.

## Discussion

Root canal preparation is a critical step in endodontic treatment, with the primary goal of maintaining the original canal anatomy while minimizing transportation, an essential factor for long-term clinical success. 29 30 Over the years, the development of NiTi rotary systems has advanced significantly to overcome the limitations of conventional hand files, such as canal perforation, instrument separation, excessive dentin removal in curved canals, and the time-intensive nature of manual instrumentation. 27

The present study aimed to evaluate and compare the canal transportation and centering ability of two NiTi rotary file systems, MiniKUT and TruNatomy, in the mandibular first molar MB root canals with 20 to 30 degrees of curvature. The two systems were compared as they are marketed and recommended for clinical use. Mandibular molars were selected for this study due to the moderate to severe curvature commonly found in their roots, particularly in the mesial canals, where canal curvature is more frequently observed. 31 The null hypothesis proposed that both file systems exhibit comparable performance in terms of canal shaping and centering. The findings demonstrated no statistically significant difference in canal transportation between the two systems ( *p* = 0.39), thereby supporting the null hypothesis.

Literature suggests multiple techniques to assess the centering ability and canal shaping efficiency of rotary instruments, including radiography, micro-computed tomography (micro-CT), longitudinal clearing, Bramante's latitudinal sectioning, and CBCT. 14 19 32 Micro-CT and CBCT are mostly used in studies to compare canal transportation and centering ability. Micro-CT has high radiation exposure and is limited to small sample sizes and high operational costs. 3 Moreover, modern CBCT systems provide sufficiently high resolution (voxel size: 0.125–0.2 mm) to accurately assess canal transportation and centering ability in extracted teeth, particularly when evaluating overall shaping patterns rather than sub-micron dentin changes. 14 Hence, in the present study, CBCT was chosen due to its noninvasive nature, reproducibility, and ability to provide high-resolution, three-dimensional analysis of the canal morphology pre- and post-instrumentation. 14 33 Using CBCT aligns the study methodology more closely with clinical imaging protocols, enhancing the translational relevance of the findings.

Both MiniKUT and TruNatomy are made from heat-treated nickel–titanium alloys, although the thermal processing differs: MiniKUT files undergo proprietary heat treatment designed to enhance flexibility and resistance to cyclic fatigue, whereas TruNatomy instruments are manufactured using a controlled NiTi metallurgical process that produces a unique slim-shank design with optimized flexibility and canal-centering ability. Both the endodontic files also differ in final taper, tip size, and cross-sectional design, which directly affect their rigidity, flexibility, and shaping outcomes. In this study, they were compared using the same CBCT-based methodology, enabling a valid and direct evaluation of their shaping performance. Several studies in the literature have explored the shaping efficiency of TruNatomy files; however, there are limited published data on MiniKUT instruments. The MiniKUT system has been reported only in two case studies, both involving 20- to 30-degree curved root canal anatomies. 15 The case reports on MiniKUT files have reported that due to the square cross-section and flexibility of these files, it is easy to remove debris from curved root canals. 15 In contrast, TruNatomy files are studied and compared in numerous studies due to their favorable mechanical properties. 9 10 18 19

At 3 mm from the apex, the TruNatomy system demonstrated slightly greater canal transportation compared to MiniKUT. This could be attributed to the increased rigidity of the instrument associated with greater tapering, reducing its flexibility and ability to conform to the natural canal curvature, especially in the narrow apical third. Consistent with previous findings, canal transportation in this region was more evident on the lingual side due to the anatomical configuration of curved mandibular canals. 9 34 Despite this observation, statistical analysis revealed no significant difference in canal transportation between the two groups. These findings align with earlier studies comparing TruNatomy with Reciproc Blue, VDW.ROTATE, ProTaper GOLD, and HyFlex EDM systems, where similar shaping capabilities were reported. 9 10 18 19

A significant difference was noted in the centering ability at the apical third ( *p* = 0.001), with MiniKUT outperforming TruNatomy. This result aligns with findings by Pérez Morales et al, who observed lower centering efficiency in TruNatomy compared to WaveOne Gold. 35 Conversely, studies by Al-Abady and Al-Zaka 36 and Alarfaj et al 12 reported superior centering ability for TruNatomy when compared with other systems, including EdgeOne Fire and ProTaper GOLD. These discrepancies might be attributed to differences in methodology or the novel material properties of MiniKUT, which remain largely unexplored in the literature.

Pearson's correlation analysis indicated a significant positive correlation at the 3-mm level in the MiniKUT group ( *r* = 0.579, *p* = 0.024), suggesting that the instrumentation technique had a greater influence on centering ability at the apical third. In contrast, no significant correlations were observed at other levels for either group, indicating consistent shaping behavior by TruNatomy at all measured levels. These findings suggest that the MiniKUT system may exhibit better performance in canal shaping at the apical third in curved canals, potentially due to differences in file design or flexibility compared to the TruNatomy system. The consistent shaping behavior of TruNatomy across all levels may be attributed to its off-centered cross-sectional design and enhanced flexibility, which help maintain the original canal curvature more effectively. 2 10 15 16 Further studies with larger sample sizes and different canal configurations are recommended to validate these observations and assess the long-term clinical implications of these two novel instrumentation systems.

The results of the current study indicate that MiniKUT files exhibited less transportation and better centering ability compared to TruNatomy files. This performance may be attributed to the file's unique square cross-section and the square angles of its four flutes. Additionally, these files are manufactured from heat-treated NiTi alloy, which is claimed to provide greater flexibility, helping to maintain canal integrity, particularly in curved canals. The present study compared two rotary file systems as they are marketed and recommended for clinical use. Although the MiniKUT procedure pack for small canals consists of three files, while the TruNatomy assorted pack contains five, this difference reflects the inherent design and packaging of each manufacturer. It is important to note that the TruNatomy system includes an orifice shaper and a glider as part of its sequence, whereas the MiniKUT system does not. These auxiliary instruments may contribute to canal enlargement and shaping outcomes. Given the ongoing advancements in NiTi alloys and file designs, further in vitro studies are necessary to evaluate the shaping efficacy of newly introduced instruments and their adherence to ideal canal preparation standards.

### Study Limitations

Despite efforts to standardize the experimental protocol, certain limitations should be acknowledged.

1. Chairside instrumentation time was not recorded, which could provide further insights into the clinical efficiency of the two systems.
2. Canal transportation was assessed only in the mesiodistal plane, while buccolingual transportation was not evaluated.
3. Due to a lack of information in the literature regarding MiniKUT files, direct comparison of the present findings with previous studies was not possible.
4. Lastly, in vitro studies primarily emphasize immediate technical outcomes rather than long-term clinical success. Future research should focus on in vivo studies, alongside evaluating operative time and other clinically relevant outcomes.

## Conclusion

Within the limitations of this in vitro study, MiniKUT and TruNatomy rotary instruments demonstrated comparable canal transportation at 3, 6, and 9 mm from the root apex, with slightly higher transportation for TruNatomy at 3 mm. Both systems exhibited statistically significant differences in centering ability across measurement levels, with improved centering observed at 9 mm compared to 3 and 6 mm. Overall, the findings indicate that both instruments provide consistent shaping performance while preserving canal anatomy, suggesting their potential suitability for minimally invasive endodontic preparation. Further in vivo studies are recommended to validate these results.

## References

[1] Akshay V A Srirekha A Reddy J Champa C Shetty A Srinivasan A Evaluation of the efficacy of TruNatomy, ProTaper retreatment, and RaCe file systems in retreatment of moderately curved mandibular molars: an *in vitro* study J Conserv Dent Endod 2023 26 04 383 387 10.4103/jcd.jcd_143_23 PMC10497093 37705543

[2] Bürklein S Hinschitza K Dammaschke T Schäfer E Shaping ability and cleaning effectiveness of two single-file systems in severely curved root canals of extracted teeth: Reciproc and WaveOne versus Mtwo and ProTaper Int Endod J 2012 45 05 449 461 10.1111/j.1365-2591.2011.01996.x 22188401

[3] Sunildath M S Mathew J George L Vineet R V Thomas P John D Canal transportation and centering ability of root canals prepared using rotary and reciprocating systems with and without PathFiles in cone-beam computed tomography-based three-dimensional molar prototypes J Conserv Dent 2021 24 03 246 251 10.4103/jcd.jcd_200_21 PMC8717843 35035149

[4] Waleed D Selivany B J Mohammad J R Debridement ability of TruNatomy, S-One Plus, and other single file systems Open Access Maced J Med Sci 2022 10(D):91 97

[5] Gergi R Rjeily J A Sader J Naaman A Comparison of canal transportation and centering ability of twisted files, PathFile-ProTaper system, and stainless steel hand K-files by using computed tomography J Endod 2010 36 05 904 907 10.1016/j.joen.2009.12.038 20416443

[6] Zupanc J Vahdat-Pajouh N Schäfer E New thermomechanically treated NiTi alloys - a review Int Endod J 2018 51 10 1088 1103 10.1111/iej.12924 29574784

[7] Topçuoğlu H S Düzgün S Aktı A Topçuoğlu G Laboratory comparison of cyclic fatigue resistance of WaveOne Gold, Reciproc and WaveOne files in canals with a double curvature Int Endod J 2017 50 07 713 717 10.1111/iej.12674 27344032

[8] Riyahi A M Bashiri A Alshahrani K Alshahrani S Alamri H M Al-Sudani D Cyclic fatigue comparison of TruNatomy, Twisted File, and ProTaper Next rotary systems Int J Dent 2020 2020 01 3.190938E6 10.1155/2020/3190938 PMC7061136 32184827

[9] Chércoles-Ruiz A Sánchez-Torres A Gay-Escoda C Endodontics, endodontic retreatment, and apical surgery versus tooth extraction and implant placement: a systematic review J Endod 2017 43 05 679 686 10.1016/j.joen.2017.01.004 28343928

[10] Eliasz W Kubiak K Poncyljusz W Surdacka A Root canal transportation after root canal preparation with ProTaper Next, WaveOne Gold, and twisted files J Clin Med 2020 9 11 3661 10.3390/jcm9113661 PMC7696375 33202555

[11] Özyürek T Yılmaz K Uslu G Shaping ability of Reciproc, WaveOne GOLD, and HyFlex EDM single-file systems in simulated S-shaped canals J Endod 2017 43 05 805 809 10.1016/j.joen.2016.12.010 28292599

[12] Alarfaj B Elsewify T El-Sayed W Eid B Canal transportation and centering ability of thermally-treated NiTi files J Inter Dent Med Res 2022 15 02 556 560

[13] Bürklein S Mathey D Schäfer E Shaping ability of ProTaper NEXT and BT-RaCe nickel-titanium instruments in severely curved root canals Int Endod J 2015 48 08 774 781 10.1111/iej.12375 25156248

[14] Hasheminia S M Farhad A Sheikhi M Soltani P Hendi S S Ahmadi M Cone-beam computed tomographic analysis of canal transportation and centering ability of single-file systems J Endod 2018 44 12 1788 1791 10.1016/j.joen.2018.09.011 30390970

[15] Buchanan L S Verbanck C L Root canal shaping in the age of minimally invasive endodontics (MIE) Endod Practice US 2023 16 01 20 24

[16] Eckstaine J Rotary Instrumentation as a First File to Length Saint Louis University 2025

[17] Hülsmann M Donnermeyer D Schäfer E A critical appraisal of studies on cyclic fatigue resistance of engine-driven endodontic instruments Int Endod J 2019 52 10 1427 1445 10.1111/iej.13182 31267579

[18] Del Fabbro M Afrashtehfar K I Corbella S El-Kabbaney A Perondi I Taschieri S In vivo and in vitro effectiveness of rotary nickel-titanium vs manual stainless steel instruments for root canal therapy: systematic review and meta-analysis J Evid Based Dent Pract 2018 18 01 59 69 10.1016/j.jebdp.2017.08.001 29478682

[19] Elsaka S E Elnaghy A M Badr A E Torsional and bending resistance of WaveOne Gold, Reciproc and twisted file adaptive instruments Int Endod J 2017 50 11 1077 1083 10.1111/iej.12728 27917513

[20] Ghavami-Lahiji M Davalloo R T Tajziehchi G Shams P Microcomputed tomography in preventive and restorative dental research: a review Imaging Sci Dent 2021 51 04 341 350 10.5624/isd.20210087 PMC8695474 34987994

[21] Purba R Sonarkar S S Podar R Singh S Babel S Kulkarni G Comparative evaluation of retreatment techniques by using different file systems from oval-shaped canals J Conserv Dent 2020 23 01 91 96 10.4103/JCD.JCD_167_20 PMC7657436 33223649

[22] Schneider S W A comparison of canal preparations in straight and curved root canals Oral Surg Oral Med Oral Pathol 1971 32 02 271 275 10.1016/0030-4220(71)90230-1 5284110

[23] Centers for Disease Control and Prevention.Best practices for handling extracted teethCDC; December 15,2023[cited September 1, 2025]. Accessed September 23, 2025 at:https://www.cdc.gov/dental-infection-control/hcp/dental-ipc-faqs/extracted-teeth.html

[24] Jaggi P Mulay S Tandale A S Jadhao R Aras S Patil R Comparative evaluation of shaping ability of TruNatomy rotary endodontic file versus different rotary endodontic file systems J Inter Clin Dent Res Org 2023 15 01 13 22

[25] Thomas A R Reddy T VK Tan J Y Analysis of shaping abilities of rotary file systems in oval canals with moderate root curvature: a randomized controlled *ex vivo* study J Contemp Dent Pract 2023 24 04 221 227 10.5005/jp-journals-10024-3471 37469260

[26] Vasava N R Modi S H Joshi C Evaluation of the centering ability and canal transportation of rotary file systems in different kinematics using CBCT World J Dental 2023 14 11 984

[27] Torabinejad M Cho Y Khademi A A Bakland L K Shabahang S The effect of various concentrations of sodium hypochlorite on the ability of MTAD to remove the smear layer J Endod 2003 29 04 233 239 10.1097/00004770-200304000-00001 12701769

[28] Gambill J M Alder M del Rio C E Comparison of nickel-titanium and stainless steel hand-file instrumentation using computed tomography J Endod 1996 22 07 369 375 10.1016/S0099-2399(96)80221-4 8935064

[29] Peters O A Schönenberger K Laib A Effects of four Ni-Ti preparation techniques on root canal geometry assessed by micro computed tomography Int Endod J 2001 34 03 221 230 10.1046/j.1365-2591.2001.00373.x 12193268

[30] Martins J N Marques D Silva E JNL Caramês J Versiani M A Prevalence studies on root canal anatomy using cone-beam computed tomographic imaging: a systematic review J Endod 2019 45 04 372 3.86E6PubMed 10.1016/j.joen.2018.12.016 30833097

[31] Faisal I Saif R Alsulaiman M Natto Z S Shaping ability of 2Shape and NeoNiTi rotary instruments in preparation of curved canals using micro-computed tomography BMC Oral Health 2021 21 01 595 10.1186/s12903-021-01961-x PMC8603484 34798874

[32] Dantas W CF Marceliano-Alves M FV Marceliano E FV Microtomographic assessment of the shaping ability of the Hyflex CM and XP-endo Shaper systems in curved root canals Eur J Dent 2023 17 03 699 705 10.1055/s-0042-1750694 PMC10569844 36220114

[33] Pasqualini D Alovisi M Cemenasco A Micro–computed tomography evaluation of Protaper Next and BioRace shaping outcomes in maxillary first molar curved canals J Endod 2015 41 10 1706 1710 10.1016/j.joen.2015.07.002 26321061

[34] Capar I D Ertas H Ok E Arslan H Ertas E T Comparative study of different novel nickel-titanium rotary systems for root canal preparation in severely curved root canals J Endod 2014 40 06 852 856 10.1016/j.joen.2013.10.010 24862716

[35] Pérez Morales M LN González Sánchez J A Olivieri J G Micro–computed tomographic assessment and comparative study of the shaping ability of 6 nickel-titanium files: an in vitro study J Endod 2021 47 05 812 819 10.1016/j.joen.2020.12.021 33549630

[36] Al-Abady A M Al-Zaka I M Evaluation of the canal transportation and centering ability of different rotary NiTi systems in simulated curved canals: a comparative study Indian J Forensic Med Toxicol 2021 15 03 1396 1406

